# Simultaneous surgery for subcondylar fracture and prominent angle of the mandible

**DOI:** 10.1186/s40902-015-0028-y

**Published:** 2015-08-28

**Authors:** Chang-Hwa Jeong, Jae-Young Ryu, Woo-Yul Lee, Hyeon-Min Kim

**Affiliations:** Department of Oral & Maxillofacial Surgery, Gachon University Gil Medical Center, 21, Namdong-daero 774 beon-gil, Namdong-gu, Incheon, 405-760 Republic of Korea

**Keywords:** Mandibular condyle, Mandibular osteotomy, Botulinum toxin

## Abstract

We experienced a patient of subcondylar fracture who had a squared contour of the lower face with prominent angle of the mandible and masseter hypertrophy. Our patient was increasingly seeking esthetic improvement of the lower third of the face. But she did not want multi-stage operations. Thus, we decided and performed a one-stage mandibular angle ostectomy with fracture management. We have a stable and esthetic result simultaneously despite fractures of the fixation plates during follow-up period, so report a case.

## Background

A prominent mandibular angle with a square face symbolizes masculinity in a viewpoint of Korean people [1]. Factors contributing to the contour of the lower face include the shape of the mandible and the bulkiness of the masseter muscles. Available treatments include from combined muscle and bone resection to injections of botulinum toxin on the masseter muscle. So ostectomy of the mandibular angle or botulinum toxin injection is performed in the many Korean people depending on a patient’s esthetic demands.

Ostectomy of the mandibular angle or management of subcondylar fracture can be performed through an intraoral approach. But intraoral approach offers limited visual field and difficulty during operation. The purpose of this article is to report a case wherein a simultaneous surgery for subcondylar fracture and prominent mandibular angle was performed intraorally.

## Case presentation

**Fig. 1:**
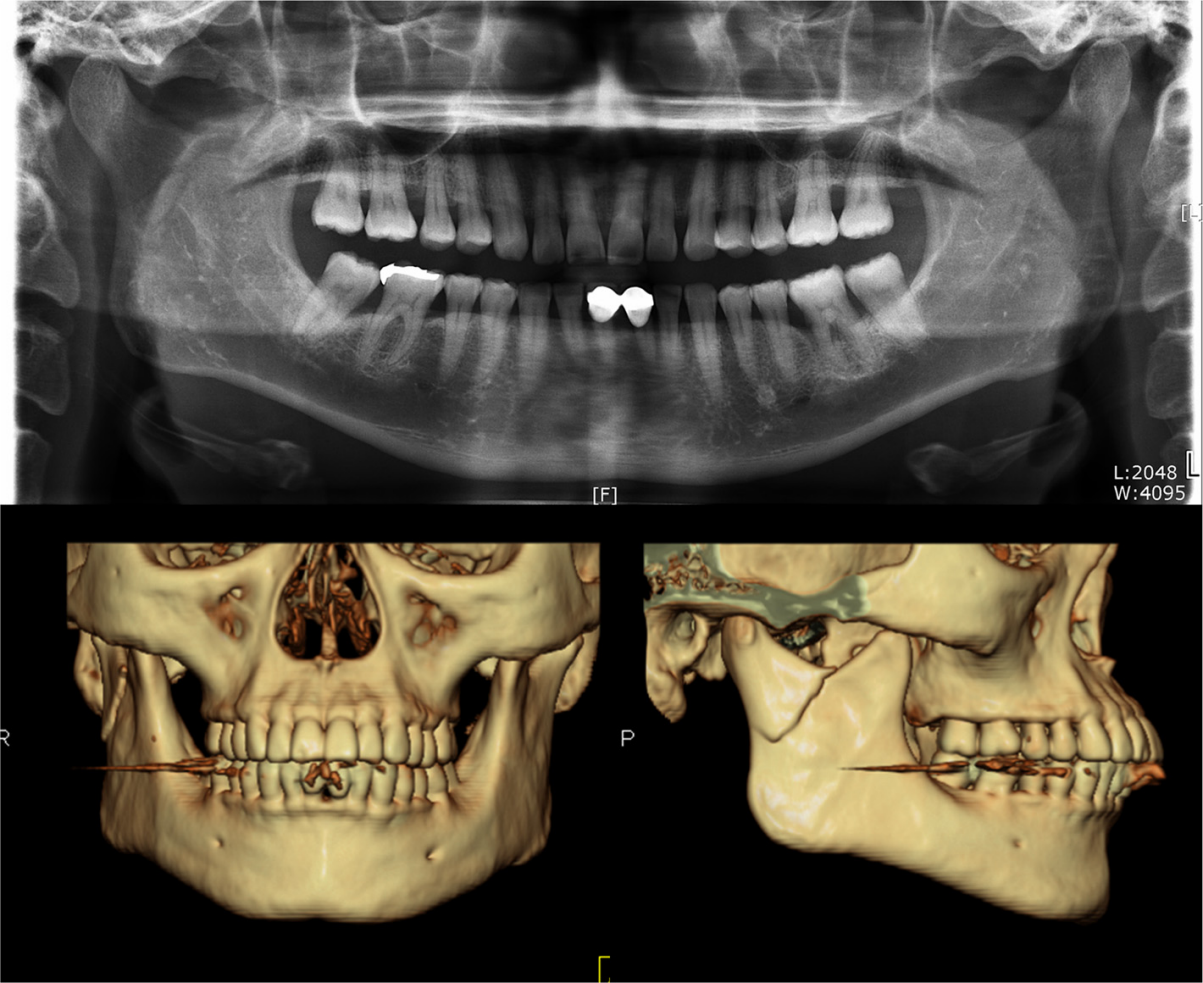
Preoperative: panoramic radiograph (*above*) and 3D-CT, frontal and right lateral view, (*below*) showing the fractured right subcondyle and prominent angle of mandible

A 38-year-old woman, victim of a family violence, came to our department with a subcondylar fracture on the right side of mandible (Fig. 1). She presented malocclusion with swelling and tenderness on the right cheek and preauricular area and was planned for surgery under general anesthesia. By the way, she complained of bilateral prominent mandibular angles and wanted angle ostectomy simultaneously. She also had the bilateral masseter hypertrophy. So we decided and proceeded with simultaneous surgery of fracture management and angle ostectomy.

**Fig. 2:**
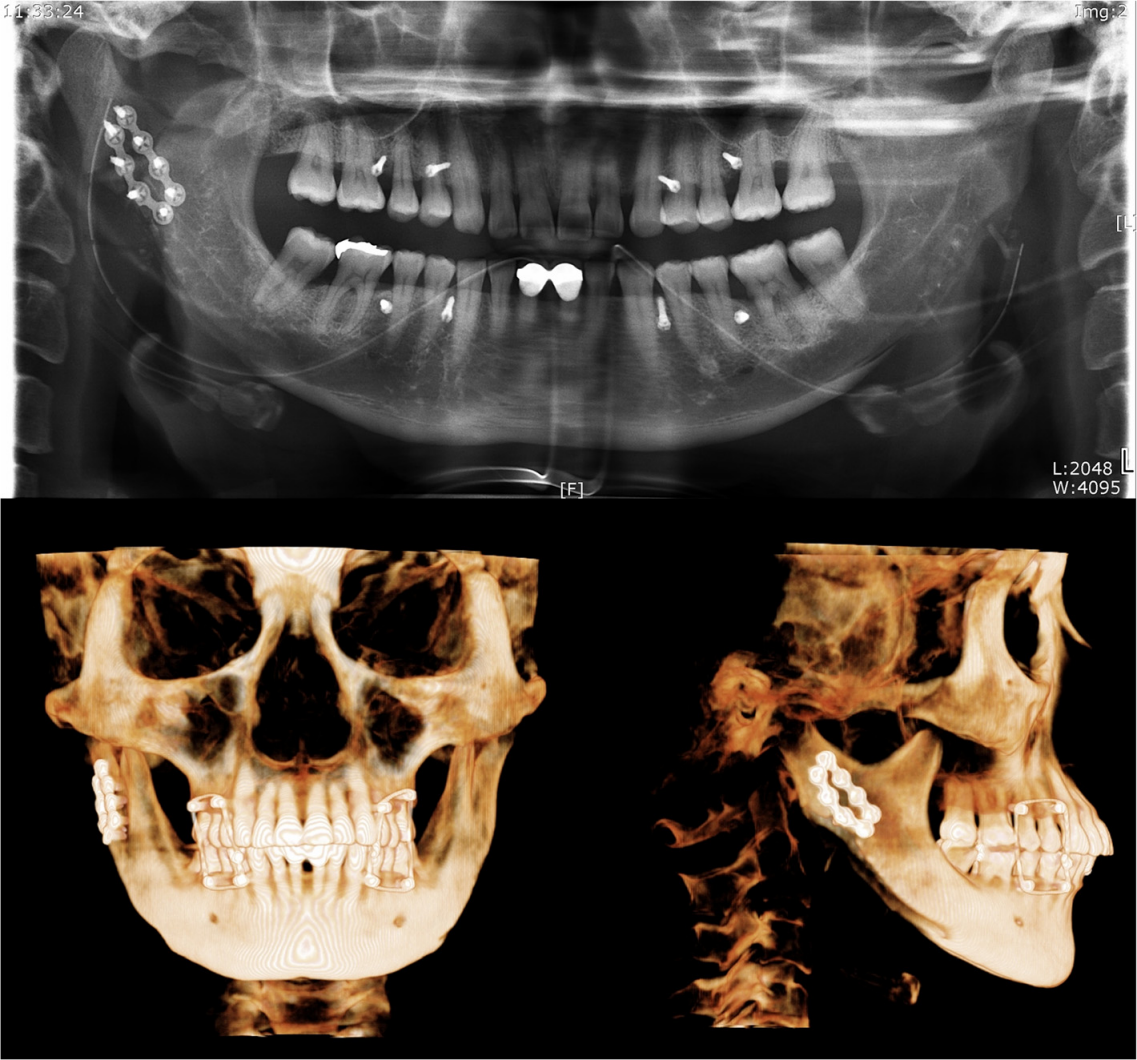
Immediate postoperative: panoramic radiograph (*above*) and 3D-CBCT, frontal and right lateral view, (*below*) showing the damage of posterior plate

**Fig. 3:**
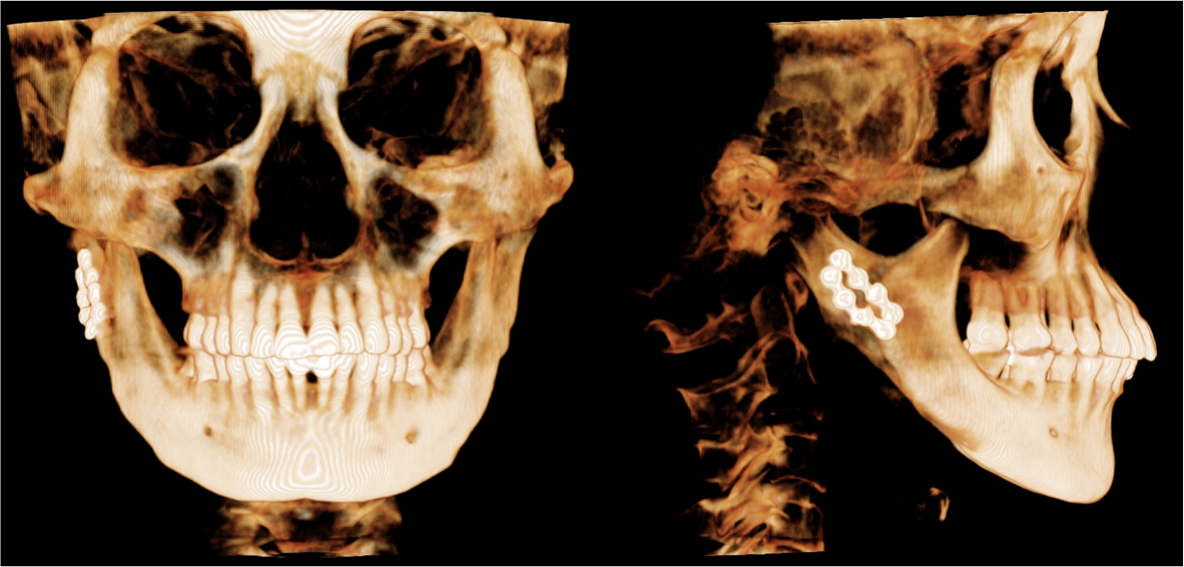
Postoperative 8-month 3D-CBCT, frontal and right lateral view, showing no distinct displacement of condylar fragment

The patient was operated under general anesthesia with nasotracheal intubation. Intermaxillary fixation (IMF) was installed using eight IMF screws during operation. Lidocaine with 1:100,000 epinephrine was infiltrated in the posterior vestibule and retromolar area of both lower sides. First, the fractured subcondyle was reduced transorally via a vestibular approach. After that, two 2.0-mm system plates and screws (Synthes Inc., West Chester, PA, USA) were used for fixation with the help of a trocar. The first plate was usually installed in the posterior border of the ramus. However, in this case, we placed the first plate in the anterior portion of the estimated ostectomy line, because the posterior border of the ramus would be resected to some extent. Then, the second plate was installed more anteriorly than the first plate. Two miniplates were positioned at the thickest part of subcondylar area in a surgeon’s preference. Then, the IMF was removed and the mouth was opened and the occlusion and mandibular movement were checked. After that, usual angle ostectomy of both sides was progressed using bur, oscillating and reciprocating saw in a condition of IMF to stabilize the mandible. In the immediate postoperative period, we recognized damage of the fixation plate. The damaged trace of the fixation plate was observed at postoperative radiograph (Fig. 2). It was assumed that the reciprocating saw was invaded to posterior plate. The patient was kept 5 days in IMF with elastics to keep the proper occlusion. After releasing the IMF, exercise was started with guiding elastics for 2 weeks. After that, only opening exercise was performed by herself for another 1 week. Then, she had a normal range of mouth opening. She had no difficulty or pain in mouth opening at the first month after the operation. But plate fractures were confirmed in a panoramic radiograph at that period. After that, we planned the closed observation without any intervention. Though more dislocated fragments of fractured plates were observed at the third month of postoperative period, there were no clinical symptoms. But the muscular strength of both masseters gradually increased, so botulinum toxin type A (BOTOX, Allergan Inc., Irvine, CA, USA) was applied at 3 months after the operation. Two 25-unit injections were applied on each masseter area of the face. Despite the fracture of plates, there was no clinical problem until 8 months after the operation and the result satisfied her esthetic demand (Fig. 3). This study was approved by the regional Ethical Review Board of the Gachon University Gil Medical Center (Certificate No.: GAIRB2015-66).

### Discussion

Because there is enough bone stock to stabilize a fixation plate, subcondylar fractures may be amenable to rigid fixation. Though there are different approaches for the fixation of mandibular subcondylar fractures, that area can be typically well visualized using instrument such as Bauer retractors, followed by anatomic reduction transorally [2]. In this case, fractured fragment displaced laterally and patient did not want to make operation scars in her face. Mandibular angle ostectomy can be also performed transorally [3]. So we chose intraoral approach. The intraoral approach for mandibular surgery offers many advantages that minimize the risks of visible scars and facial nerve injury. But the limitation of that approach is a narrow surgical field, which makes it difficult to view the operative site directly. So there is possibility of surgical and esthetic complications. In this case, we experienced the fractures of fixation plates.

Osteosynthesis of subcondylar fractures with two straight plates has been shown to be a suitable method that withstands the functional loading transmitted to that area [4, 5]. In this case, we also used two 2.0-mm system plates and screws. Because the estimated area of angle ostectomy was overlapped with reduction and fixation area, posterior object among two plates was located more anteriorly than the usual position. Anatomical reduction is the most important thing in the operation of fracture management. So, reduction and fixation were progressed before angle ostectomy. After fixation, usual ostectomy of mandibular angle was performed under the plate fixation and IMF state to make the mandible stable. Because that situation is more similar and stable condition in shape than in displaced state, the operator could be more familiar for angle ostectomy. But, it was assumed that fixation plate was damaged by the reciprocating saw during angle ostectomy. Distortion of plate shape could be observed on immediate postoperative radiograph. Even though the operator paid particular attention to ostectomy, plate damage occurred on the account of limited operation field of vision. Immediately after the operation, there was no clinical problem such as malocclusion despite the damage of a plate. After that, individual protocol such as postoperative IMF (for 5 days) and elastic training (for additional 2 weeks) was carried out.

The fractures of two fixation plates were observed in panoramic radiograph at the first postoperative month. Posteriorly positioned plate was already broken during the ostectomy as confirmed from the immediate postoperative radiograph. Moreover, it is presumed that vibration of sawing for ostectomy could be an additional reason for weakening of plates. Though fixation plates were damaged, proximal fragment of fracture site was not displaced. There were also good occlusion and recovery of mouth opening, so we planned the closed observation without any intervention, because non-surgical and conservative management can be recommended when there is no displacement of subcondylar fractures [6, 7]. Limited mouth opening and relatively soft diet were instructed to patient for one more month. Despite the fracture of plates, there was no more displacement of subcondylar fragment or clinical problem until 8 months after the operation. Though there was no clinical problem in this case, stable fixation of fracture site is important. So if simultaneous treatment is necessary such as this patient, we suggest that reduction and fixation first then, do the ostectomy after the removal of the existing fixation plate. So after finishing the ostectomy, fixation plate will be repositioned to the original fixation site, like when using reconstruction plate for segmental mandibulectomy. This method will help in minimizing plate and screw weakening.

Over time, muscular strength of both masseters gradually increased, so injection of botulinum toxin type A was considered. Yan et al. measured the maximal bite force, maximal opening of the mouth, and maximal protrusion before and after curved osteotomy of the mandibular angle to evaluate the effect of mandibular resection on oral physiological function [8]. They found that function was partially restricted during the early postoperative stage but was recovered by 3 months postoperatively. So, botulinum toxin was applied at 3 months after the operation in our patient for the reduction of masseter muscular power and making a more slender face. Intramuscular injection with botulinum toxin leads to the reduction of the thickness of the masseter, without serious side effects [9].

## Conclusions

We experienced a case wherein simultaneous surgery for subcondylar fracture and prominent mandibular angle was performed intraorally, and fracture of fixation plates happened after the operation. Despite of a limited surgical approach and fracture of fixation plates during and after the operation, there was no clinical problem and displacement of fractured fragment until the postoperative 8 months. The patient was also satisfied in the esthetic aspect. We operated fracture management and angle ostectomy simultaneously; then, we had a satisfactory result with the postoperative proper management.

## Consent

Written informed consent was obtained from the patient for publication of this case report and any accompanying images. A copy of the written consent is available for review by the Editor-in-Chief of this journal.
